# Response of Bare and CFRP-Retrofitted Multi-Column Piers Under Post-Fire-Coupled Vehicle Collision and Air Blast

**DOI:** 10.3390/ma18071449

**Published:** 2025-03-25

**Authors:** Qusai A. Alomari, Daniel G. Linzell, Mubarak F. Abu Zouriq

**Affiliations:** 1 Midwest Roadside Safety Facility, University of Nebraska-Lincoln, Lincoln, NE 68583, USA; 2 Department of Civil and Environmental Engineering, University of Nebraska-Lincoln, Lincoln, NE 68586, USA; dlinzell@unl.edu (D.G.L.); mabuzouriq2@huskers.unl.edu (M.F.A.Z.)

**Keywords:** bridge piers, reinforced concrete columns, fire, vehicle collision, air blast, multi-hazard, structural response, CFRP retrofitting, finite element analysis, LS-DYNA

## Abstract

Numerous catastrophic events, including fire, vehicle collisions, and air blasts, have highlighted the significance of examining bridge performance under multi-hazard scenarios. While these hazards cause extensive damage, the loss of life, and drastically impact economies, limited attention has been devoted to study the behavior of bridge structural elements under such extreme demand combinations. Hence, comprehensive research to understand the resiliency of bridges and their response to combinations of fire, vehicular impact, and air blast is warranted so that effective retrofitting techniques can be developed and design recommendations be made. To address this research gap, present investigations utilized previously validated finite element (FE) models in LS-DYNA to study the structural behavior of two-, three-, and four-column piers under post-fire medium truck collision and subsequent air blast. The response of multi-column piers was quantified and evaluated based on damage propagation, failure patterns, and permanent deformation sets. The effectiveness of selected retrofitting techniques that employed carbon-fiber-reinforced polymers (CFRPs) to mitigate damage was investigated. Study findings enhance current understanding, provide valuable insights, and can ultimately be used to ensure safety and improve the structural integrity of bridge piers under coupled vehicle collision and air blast following fire exposure.

## 1. Introduction

During their service lives, highway bridges are vulnerable to a wide spectrum of extreme hazards, including vehicle collisions, earthquakes, hurricanes, floods, explosions, and fires [1]. Located adjacent to roadways, and not adequately protected, bridge piers are susceptible to errant vehicle collisions. As highlighted in many research studies and surveys, vehicle collision is a primary cause of bridge collapse in the U.S. A total of 1062 bridge failures in the U.S between 1980 and 2012 were collected and indicated that vehicle collisions are marked as the second potential cause of bridge failure [2]. Another survey carried out on 500 bridges in the U.S between 1989 and 2000 demonstrated that approximately 20% of reported bridges failed under vehicle collision [3]. The National Transportation Safety Board (NTSB) revealed that among all vehicle categories, more than 1000 truck impacts are recorded annually, causing severe damage to the bridge structure or necessitating a complete or partial replacement [4].

It is highly recognized that bridge multi-hazard events, which are typically referred to as vehicle collisions correlated to other cascading extreme demands, such as air blast and fire, are assumed to be rare events. Specifically, bridge design standards and numerous research studies have extensively examined bridge performance and proposed design and analysis methods under individual extreme events, while limited attention has been devoted to the study of the complex effects of simultaneous or subsequent multiple hazards. However, multi-hazard events that involve vehicle collision, air blasts, and fires are considerably increasing in frequency and leading to catastrophic failures, adverse economic overburdens, and the loss of lives and properties. These critical consequences have been highlighted by many recent incidents that involved vehicle collision, air blast, and fire. In a recent incident, a truck tanker crashed into a bridge pier in Philadelphia in June 2023, causing a significant portion of the bridge substructure to collapse [5]. It was reported that, in addition to the truck collision, this tragic collapse incident was also attributed to explosions in underground mains and subsequent intensive fires. As a result, studies that further examine bridges under various combinations of the aforementioned hazards are still needed to better understand the corresponding structural behavior and to propose potential in situ retrofit schemes and design and analysis procedures.

Given the rapid development of urban transportation systems and the increased transportation of highly flammable goods, the rate of bridge fire incidents significantly increased over the past few years in the U.S [6,7]. Hence, fire hazard is being recently treated as a potential extreme demand acting independently or within a multi-hazard event [7,8,9]. Unlike impact and blast, fires in open spaces, like highway bridges, are completely ignored by relevant bridge design codes and specifications utilized in the U.S. [10]. Consequently, bridge fires pose a major challenge for bridge designers and practitioners. As noted by Giuliani et al., bridge design codes overlook fire in structural design and analysis provisions due to challenges associated with accurately defining its characteristics [9]. More specifically, uncertainties persist with respect to critical factors influencing fire intensity, including heating duration, fire origin and location, flame height, heat release rate, and ambient conditions [11]. Several studies indicated that bridge fires are caused by petrochemicals, overturned vehicles, highly flammable fuels carried by tanker trucks, electrical problems, stored materials, and forest fires or arson [8,9,12]. Each of these fires has a distinct heat release rate, resulting in variations in the time required to reach peak temperatures. As a result, a gap exists in understanding how bridges and bridge structural components perform during and after a fire.

Substantial efforts were dedicated to study the performance of isolated bridge columns under vehicle collision acting independently or with fire and air blast earlier research project stage [13,14,15,16]. While one stage aimed at deriving an empirical equation to estimate the Equivalent Static Force (ESF), which is widely used to design bridge columns under vehicle collisions, another project phase focused on evaluating bridge column performance under various sequences of vehicle collision, air blast, and fire. Particularly, two possible fire exposure scenarios, with fire happening before or after a coupled vehicle collision and air blast, were investigated. While the sequence of collision, air blast, and then fire is more obvious, the case in which impact and blast followed a fire exposure was deemed more critical. This specific loading sequence was deliberately selected to see if columns could resist dynamic extreme events after being thermally degraded. This scenario was also essential to study the effectiveness of post-fire repair columns and to explore how any potential performance enhancement under high-strain rate loading can be achieved. Several previous research studies underscored this need, indicating that the response of fire-damaged critical reinforced concrete (RC) structural units, such as bridge pier columns, should be assessed under high loading rates [17,18]. This is mainly because exposing concrete to elevated temperatures significantly reduces its dynamic compressive strength, which considerably influences post-fire responses and requires reasonable performance revaluation of exposed RC structural elements [17,18,19]. For highway bridges and their structural components, studying the post-fire performance is justified if these structural elements survived fire and maintained adequate capacity to keep the element in service, with or without repair. Several research studies have examined the performance of RC structural elements subjected to fire exposure in conjunction with high loading rates caused by impact or air blast demands [20,21,22,23]. These studies did not, however, investigate RC bridge pier column performance under combined effects of vehicle impact and air blast after fire damage.

Given their strength-to-weight ratio, fiber-reinforced polymers (FRPs) have emerged as an effective method to repair fire-damaged RC columns to enhance their structural performance [24]. While their initial cost might be higher than other retrofitting methods, such as steel or concrete jacketing, the reduced labor and installation time, ease of implementation, and high durability make FRP composites a widely preferred practical option in bridge retrofitting applications [24,25,26]. Typically, utilizing FRP composites to retrofit structural elements can be implemented in multiple techniques. A widely used method refers to the near-surface-mounted (NSM) retrofit scheme, where FRP strips, robes, or bars are bonded to the surface of the structural member or installed at a specific depth using special bonding agents. Another common approach is known as the externally bonded retrofit technique in which FRP sheets are adhesively attached to the external surface of the damaged structural element. In many other circumstances, hybrid retrofitting methods are implemented to achieve better performance under complex loading conditions. In these retrofit approaches, a combination of NSM and EB FRP composites are utilized. Typically, multiple factors, such as damage extent, the type of structural element, and imposed loads, control the selection of the retrofitting scheme. For instance, a combination of NSM FRP bars and EB FRP sheets are frequently used to enhance the flexural and shear capacities of RC beams. Given that concrete compressive strength increases with increasing the confinement pressure, wrapping RC columns using EB FRP sheets is typically implemented to enhance their axial load capacity [27].

Given their capabilities in improving the overall structural capacities and resiliency under various loading scenarios, several research studies have been conducted to investigate the efficiency of FRP-retrofitted structural elements under individual impact or blast [28,29,30,31,32,33]. It was demonstrated that incorporating FRP in strengthening structural components largely improved their response to imposed demands. On the other hand, utilizing FRP laminates in repairing fire-damaged structural units has received significant attention over the past two decades. Numerous experimental and numerical research studies have indicated that utilizing various FRP retrofitting schemes notably performed well in restoring structural capacities and integrities of structural components after being exposed to various fire intensities [34,35,36,37,38,39]. Nevertheless, none of the cited research studies have explored the behavior of bare fire-damaged and FRP-repaired RC bridge columns under vehicular impacts and air blasts.

Building upon prior studies, the current study advances the current knowledge in the field of bridge multi-hazards by incorporating the effects of fire into subsequent impact and blast, which has not been previously explored. While the performance of bridge systems has been extensively examined under individual extreme events or other combinations [17,18,20,23,33,40,41,42], previously highlighted catastrophic incidents indicate that studying the selected complex sequence of hazards is needed and fills a critical knowledge gap. As a result, the present study examined the effectiveness of in situ retrofitting schemes utilizing validated finite element (FE) models of intact and fire-damaged multi-column piers in LS-DYNA [14,15,40]. Despite its computational cost requirements, the FE modeling adopted in this study ensures a more realistic and accurate representation of real-world problems. For instance, full soil and air domains were utilized in the developed model to better simulate the blast wave propagation and soil–structure interaction. In addition to damage assessment, the study proposes in situ retrofitting CFRP schemes that are not only used to repair fire-damaged columns but also to enhance their capacity under critical combinations of vehicle impact and blast. The effectiveness of using two retrofit techniques that adopt CFRP composites to repair fire-damaged RC columns prior to coupled vehicle collision and air blast was studied. Four CFRP wrapping schemes along with one hybrid technique that employed EB CFRP wraps and NSM CFRP bars were examined. Following fire exposure and the implementation of the CFRP retrofit, the performance of the bare and repaired structural system was compared and assessed under combined single-unit truck impact and air blast. This comprehensive approach, which includes investigating damage progression and post-fire retrofit, is unprecedented in the literature. While the study findings provide useful insights into bridge performance under multi-hazards, it also establishes the ground base for subsequent research that focuses on enhancing bridge resilience under these extreme demands.

## 2. Numerical Modeling

Three-dimensional, nonlinear, finite element models of bare fire-damaged and CFRP-retrofitted, two, three, and four multi-column piers, their foundations, and surrounding air and soil domains were modeled in LS-DYNA. This section details the numerical modeling approach utilized to develop the structural system.

### 2.1. Element Formulation

Different element formulations were used to model the structural components and surrounding air and soil. For all structural elements, concrete was represented by 8-node solid elements with single integration point and constant stress, while a 2-node, *Hughes–Liu* beam element was adopted to model steel reinforcement with a cross-section integration formulation. CFRP sheets were modeled using 4-node shell elements, *Belytschko-Tsay* formulation in LS-DYNA. To minimize the effect of hourglassing, a *Flanagan–Belytschko* stiffness-based hourglass control with a coefficient of 0.05 was used [43]. To accurately simulate blast wave propagation, soil and air domains were modeled using multi-material arbitrary Lagrangian–Eulerian (MM-ALE) solid elements with a viscous hourglass control type with a coefficient of $1\times 10^{-6}$ [44].

### 2.2. Material Models

#### 2.2.1. Concrete and Steel Reinforcement

Concrete was modeled using *Continuous Surface Cap Model* (CSCM) material (MAT159) in LS-DYNA, which accounts for three-dimensional yield strength, softening and hardening behavior, strain rate effects, and the damage accumulation of concrete under dynamic loading conditions. The model was first established by the FHWA and used in the current study due to its superior performance in simulating concrete behavior under dynamic loads and high strain rate conditions [45,46]. Furthermore, MAT159 was validated against a series of experiments of RC structural elements under a wide spectrum of impact and air blast loading conditions and demonstrated good performance [42,47,48,49]. The model can accurately reproduce the mechanical behavior of concrete by using the minimum number of input parameters, namely, mass density, unconfined compressive strength, and maximum aggregate size [48]. Damage accumulation is represented by a scalar parameter ranging 0 to 1 updated at every time step during the analysis, where 0 corresponds to no damage and 1 to a complete loss of stiffness and strength [43]. To simulate concrete spalling, the erosion coefficient (ERODE) was used. In accordance with previous research, this parameter was set to 1.10, for which highly stressed elements are eroded when they sustain strain values that exceed 10% of the maximum principle strain [43,45,50].

The *Piecewise Linear Plasticity* material model (MAT24) was used to model steel reinforcement. This elastoplastic constitutive model is widely employed in impact and blast analysis due to its simplified input and proven accuracy and capability to account for strain rate effects [43]. In addition to defining the elastic modulus, mass density, and yield strength, MAT24 allows for defining the plasticity curve to better simulate plastic behavior. The model also incorporates a simplified failure criterion that limits the maximum effective plastic strain and allows for highly stressed elements to erode once this specified strain value is exceeded. The current study adopted a 12% failure strain following related studies [40,51]. Moreover, strain-rate effects were considered, where Cowper and Symonds coefficients, *C* and *P*, were set to 40 and 5, respectively, as recommended by Murray et al. [44,45]. Table 1 summarizes the concrete and steel reinforcement properties used in this study.

#### 2.2.2. Soil, Air, and Explosive

As mentioned earlier, the current study integrates soil and air volumes with the structural system to better represent blast wave propagation and soil–structure interaction. The soil was simulated using *FHWA Soil* (MAT147) material [43]. This model was shown to be numerically stable when severe mesh distortion under impact and blast loading is anticipated [52]. This FHWA model employs a smooth hyperbolic yield surface in conjunction with a first-order Mohr–Coulomb failure criterion, where failure is defined by the interaction of shear and effective normal stresses exceeding the material’s failure envelope [53]. Table 2 lists the soil properties used in this study selected following previous research [54,55].

The surrounding air domain was modeled using LS-DYNA’s *Null* material model (MAT009) and incorporating the *Linear Polynomial* equation of state (EOS) available in LS-DYNA [43]. Air properties and EOS parameters are presented in Table 3. The *High Explosive Burn* material model (MAT008) was utilized to model a TNT explosive charge [43]. Compared to other models, this particular model was selected due to its accuracy in simulating detonation and blast wave propagation and compatibility with the MM-ALE approach [56]. In addition to the material model, the *Jones–Wilkins–Lee* (JWL) EOS was used to define the pressure–volume–energy relationship of the detonation products. It is worth noting that a spherical charge shape was located within the air volume at a specified distance, selected to achieve the desired blast load intensity. That was accomplished by incorporating *Initial Volume Fraction Geometry* and *Initial Detonation* keywords in LS-DYNA [43]. The TNT explosive material properties and EOS constants are listed in Table 4.

#### 2.2.3. CFRP

LS-DYNA’s *Enhanced Composite Damage model* (MAT54) was employed to model the CFRP sheets. This nonlinear material model is widely used for simulating the dynamic response of composite materials under high-strain loading conditions, such as impact and blast conditions [57,58,59]. This material model accounts for tensile, compressive, and shear failure mechanisms of both fibers and matrix of CFRP composites using the Chang–Chang failure criterion [43]. The model allows for post-stress degradation, which leads to several failure mechanisms, including matrix cracking and delamination. It also incorporates anisotropic material characteristics, enabling an accurate simulation of CFRP polymers under complex loading conditions. The number of CFRP sheet layers and the associated integration points were defined through the *Part-Composite* option in LS-DYNA, through which a more computationally cost-effective analysis can be achieved [60]. The CFRP material properties used in this study were obtained from the SikaWrap-301C manufacturer data sheets for CFRP material and approved by the Texas Department of Transportation (TxDOT), as presented in Table 5 [61]. Similar to steel reinforcement, MAT24 was used to model CFRP bars, with a mass density of 1.6 $g/{cm}^{3}$, yield strength of 2800 MPa, Modulus of Elasticity of 155 GPa, and failure strain of 1.8%.

### 2.3. Fire, Vehicle Collision, and Air Blast Modeling

#### 2.3.1. Fire

**Figure 1:**
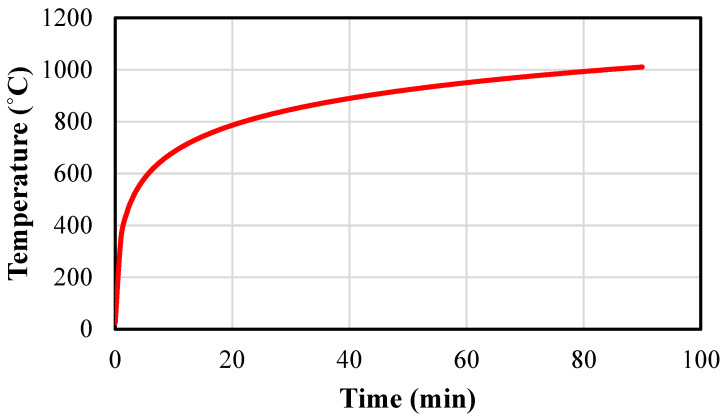
ISO-834 standard fire curve.

While LS-DYNA’s explicit solvers are commonly used to simulate structural behavior under dynamic events, the quasi-static nature of fire loads necessitates utilizing implicit solvers. Several research studies demonstrated that implicit solvers in LS-DYNA can be effectively used to simulate problems that involve heat transfer and non-linear structural-fire analysis [62,63,64]. Hence, the present study employed LS-DYNA’s static solver to simulate the structural response of bridge piers under fire exposure. A previous stage in association with the current research project was devoted to develop and validate the fire modeling approach [13,15,16]. While different fire curves are commonly employed to simulate the variation of temperature over time, ISO-834 was utilized in the current study [65]. This curve was chosen due to its widespread use in fire-induced impact and blast numerical and experimental studies. As shown in Figure 1, the ISO-834 standard fire curve simulates low-intensity fires, characterized by a slower temperature rise rate when compared to other high-intensity fire curves, such as hydrocarbon and RTZ curves. While these high intensity fire curves may be representative of real-word petrochemical fires, other fire sources, including ignitable stored materials, electrical malfunctions, construction-related fires, or arson, are typically associated with lower fire intensities [8]. Several research studies have shown that RC bridges and their structural elements are highly vulnerable to complete collapse or failure when exposed to high-intensity fires [7,66,67]. In particular, the use of more sever fire models could result in premature failure, preventing a comprehensive assessment of retrofit effectiveness and post-fire impact and blast performance. Therefore, the ISO-834 fire curve was deemed appropriate for this study, as it allows for the evaluation of bridge piers that do not completely fail under fire and remain potentially repairable or capable of retaining acceptable levels of structural integrity and capacity to resist the coupled vehicle collision and air blast [21,56,65,68,69]. To define temperature-dependent material properties and the variation of the coefficient of thermal expansion for concrete and steel reinforcement, *Thermal Isotropic TD* (MAT-T03) and *Thermal Expansion* (MAT-000) material models were adopted, respectively [43]. It is important to mention that all material characteristics associated with temperature were taken from Eurocode 2, Part 1–2 [70]. More details on fire modeling and the multi-stage modeling approach are available elsewhere [13,15,16,71].

#### 2.3.2. Vehicle Collision

**Figure 2:**
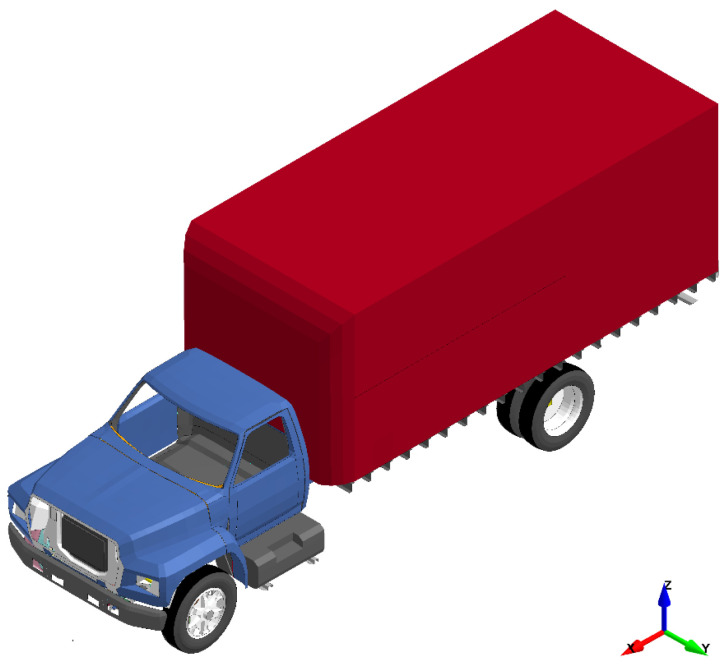
Ford F800 Single-Unit Truck (SUT), FE model.

As shown in Figure 2, the numerical model of a Ford F800 Single-Unit Truck (SUT) with a curb mass of 8175-kg was used in simulating vehicle impact on bridge piers, which originally developed by the National Crash Analysis Center (NCAC) and further refined and validated the National Highway Traffic Safety Administration (NHTSA) [72]. This high-fidelity model was selected due to its capability to mimic actual impact events, highlighted by the extensive efforts of validation against real-world full-scale crash tests. The model was designed to impact the bridge pier at a speed of 120 km/h, which is a common maximum speed limit on most rural highways in the United States.

#### 2.3.3. Air Blast

The explosive intensity is usually represented by a quantity termed scaled distance (Z), which correlates the weight of the explosive charge and the distance between the detonation origin and the target object. As per the National Cooperative Highway Research Program (NCHRP) report 645, bridges should be analyzed for Z ≤ 0.6 m/kg^1/3^ [73]. Hence, the current study considered a blast charge with Z = 0.25 m/kg^1/3^, which refers to the TNT equivalencies associated with terrorist attacks that target structures as outlined by the Federal Emergency Management Agency (FEMA) [74].

### 2.4. Model Coupling and Contacts

According to previous studies, coupling between steel and CFRP reinforcement and surrounded concrete was simulated using the *Constrained-Lagrange-in-Solid* keyword in LS-DYNA assuming perfect bonding [27,40,45]. Similarly, the same coupling approach was employed to constrain the structural system with the surrounding soil and air volumes. It is worth noting that all pier columns were fixed at the base and integrated to the pier cap at the top, as per the AASHTO-LRFD bridge design recommendations available in Section 11.2 [10].

Contact between the SUT and the structural system was simulated using the *Automatic Surface-to-Surface* contact algorithm in LS-DYNA [43]. This algorithm correlates the contact force to the total penetration of nodes lying on the interacting surfaces at each time step. To ensure an accurate representation of the impact event, a static friction coefficient of 0.30 and a contact softness factor (SOFT = 1) were used following previous studies [40,43,72]. A spherical TNT explosive charge was modeled using *Initial-Volume-Fraction-Geometry* in LS-DYNA. Additionally, the *Boundary Non-Reflecting* command was used to address the computational instabilities resulting from blast wave propagation.

The behavior of the bonding agent that is used to bond CFRP to the pier column periphery was explicitly modeled using the *Automatic-Surface-to-Surface-Tiebreak* contact type [43,57,59,75]. In this contact type, failure is set to occur whenever the failure criterion given in Equation (1) is satisfied. In this equation, $\sigma_{n}$ and $\sigma_{s}$ refer to the tensile normal and shear stresses, respectively, and *NFLS* and *SFLS* correspond to the tensile normal and shear failure stresses, respectively. As recommended in the open literature, an *NFLS* of 32 MPa and an *SFLS* of 29.4 MPa were adopted in the current study [57,75,76].

(1) $${\left(\frac{\left|\sigma_{n}\right|}{NFLS}\right)}^{2}+{\left(\frac{\left|\sigma_{s}\right|}{SFLS}\right)}^{2}\geq 1$$

In the structural-fire analysis stage, the *Control Solution* command in LS-DYNA was used to set the coupled thermal–structural solution. In the *Control Thermal Solver* option, a *Symmetric Direct Thermal Solver* (SOLVER = 11) was implemented to enable the nonlinear thermal analyses, where material properties were evaluated based on the average temperature of each element (PTYP = 2). To control the maximum temperature change between time steps, a variation threshold of 5 °C was defined through the *Control Thermal Time Step* keyword.

### 2.5. Loading Sequence

Given the multi-time-scale problem that comprises fire exposure followed by a combination of vehicle collision and air blast examined herein, a multi-stage modeling technique was utilized in accordance with previous relevant studies [11,18,23,68,77,78,79]. In this modeling approach, results from the structural-fire analysis modeling stage are incorporated with the subsequent impact and blast analysis stage. More specifically, three-dimensional heat transfer and structural-fire analyses are initially performed; then, the resulting structural response characteristics, including thermal stress and strain, geometric imperfections, and eroded elements, are carried over and used to define the initial conditions in the consequent structural analysis stage under impact and blast loading using LS-DYNA’s *Interface Springback* command [43]. As recommended by previous studies, fire was simulated as a boundary conditions, where *Initial Temperature Set* and *Boundary Temperature Set* control cards were implemented to set the initial temperature of the system and to simulate temperature variation on the exposed column surface, respectively [43,78].

**Figure 3:**
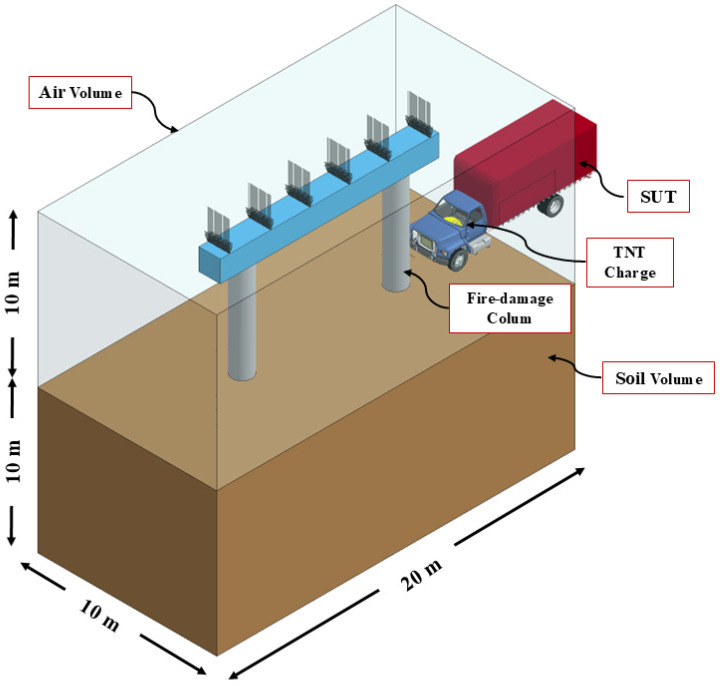
Representative FE model of two-column pier.

Building upon findings from previous research project phases, one exterior pier column was initially subjected to 90 min fire exposure before undergoing the vehicle collision and air blast loadings [13,15,16,40,51,71]. This specific fire duration was selected in accordance with previous research studies and was deemed appropriate for the present study [11,66,68]. Following fire exposure, two scenarios involving bare and CFRP-retrofitted pier columns were examined. In the subsequent impact and blast analysis stage, SUT moving at 120 km/h impacted the target pier column followed by the detonation of the TNT explosive charge. Furthermore, superstructure dead loads were considered in all analysis stages and applied through *Load-Body-Z* and *Set-Node* keywords in LS-DYNA [43]. The magnitude of the applied nodal load was set to 6% of the column’s nominal capacity and applied along the top of the cap. To reiterate, this loading sequence and corresponding fire exposure duration, impact speed, and explosive intensity were deliberately selected in the current investigation and represent the most critical scenario, as reported elsewhere [13,40,71]. Representative two-column pier is shown in Figure 3.

### 2.6. Model Validation

The accuracy of the developed FE models and utilized modeling approach was explored by comparing simulation results against publicly available individual impact and blast tests and tests that involved fire and CFRP retrofitting. In association with this validation effort, three experimental tests, including a drop hummer impact on an RC beam; a reduced-scale blast on an RC column; and the residual capacity of a fire-exposed RC column were initially utilized to validate the model performance under impact, blast, and fire loading scenarios [28,67,80]. Following this validation stage, two fire-induced impact and blast tests were used to validate model robustness under combined loading scenarios [21,23]. Moreover, the process of validation encompassed two additional tests of the CFRP-retrofitted RC column subjected to pendulum impact and the CFRP-retrofitted RC slab under near-field blast load [33,81]. Throughout this extensive validation work, the developed FE models and the corresponding modeling approach were deemed accurate relative to test results, demonstrating their capability to effectively reproduce the structural response under various combinations of the extreme events studied herein. The results from this thorough validation studies have been published by the authors of this paper, and more details can be found elsewhere [71,82].

## 3. Numerical Studies

### 3.1. Prototype Multi-Column Pier

**Figure 4:**
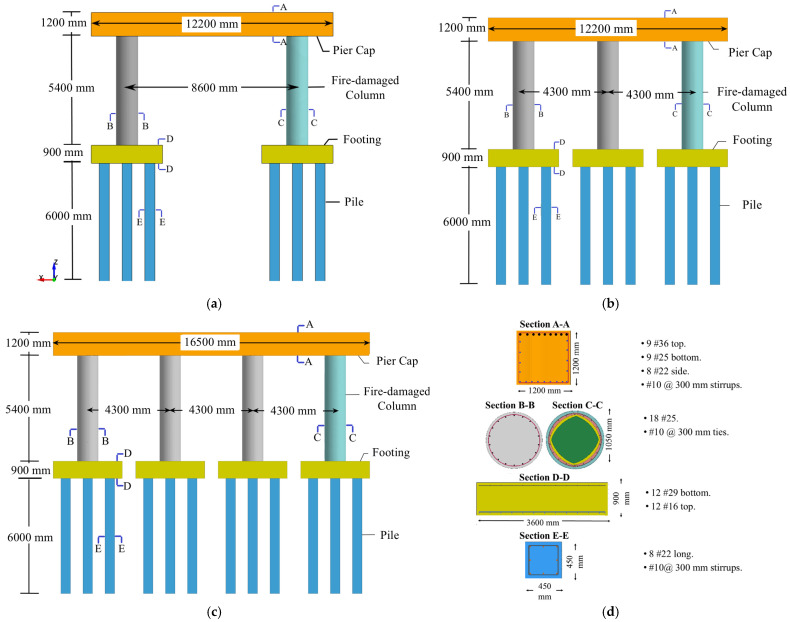
Multi-column piers: (**a**) two-column pier; (**b**) three-column pier; (**c**) four-column pier; (**d**) design details.

Following previous research project phases [83], investigations presented in this paper included two-, three-, and four-column piers. While the four-column pier was taken from a FHWA design example [84], the two- and three-column pier designs were achieved by removing supporting columns from the four-column pier system. In particular, all design variables were consistent in all configurations; however, cap length and column spans were reduced in the two- and three-column piers. While this decision was deliberately made to study various bridge pier configurations, the two- and three-column piers meet the design requirements reported in the AASHTO-LRFD bridge design specifications [10]. For all piers, columns are 5400 mm high and 1050 mm in diameter. A reinforcement ratio of 1% was achieved via using 18 No. 25 steel reinforcement bars, while No. 10 ties spaced at 300 mm were utilized. More details on pier designs are depicted in Figure 4. It is worth mentioning again that, for each pier configuration, one exterior column was assumed to be exposed to fire prior to impact and blast.

### 3.2. Response of Bare Multi-Column Pier

Simulation results are presented in this section to explore the structural performance of bare fire-damaged bridge pier under the combined vehicle collision and subsequent air blast. The most critical load combination, which encompassed a 90 min fire exposure, SUT impact at 120 km/h, and air blast with a scaled distance of 0.25 m/kg^1/3^, was used throughout this study. While exposing all columns to fire is a more conservative approach, this study considered exposing only the exterior pier column to fire and vehicle collision. This decision was purposefully made assuming the fire intensity decreases with distance from the fire’s origin, which is assumed to be concentrated around the outermost pier column. This decision was supported by recently published research that examined bridge fire models [68]. Accordingly, results from structural-fire and coupled vehicle collision and air blast analysis stages are outlined in the sections below.

#### 3.2.1. Structural-Fire Response

As mentioned earlier, the first analysis stage encompasses thermal-structural analysis, during which the temperature distribution along the column height and across its cross section is estimated based on heat transfer. While the peak temperature of approximately 665 °C was recorded at the surface of the column, temperature gradually decreased moving towards the core of the column. Given this temperature distribution, strength degradation was considered by dividing the column into layers each characterized by a unique temperature and was assigned a specific reduced strength in accordance Eurocode 2 [70]. More details on this approach can be found elsewhere.

**Figure 5:**
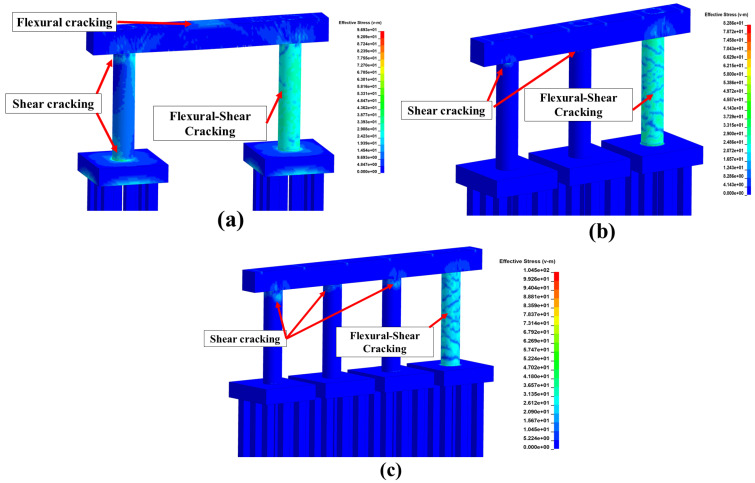
Thermal stress distribution and crack propagation: (**a**) two-column pier; (**b**) three-column pier; (**c**) four-column pier.

In addition to the mechanical strength reduction, the distribution of thermal stresses caused by exposing the exterior pier column to the selected fire is illustrated in Figure 5. As illustrated in this figure, fire exposure is attributed to flexural-shear cracks distributed over the column height. This observation matched experimental testing results reported elsewhere [39]. Irrespective of the number of columns, shear cracks have propagated into the pier cap. As expected, the two-column pier experienced more damage relative to the three- and four-column piers due to its reduced redundancy, with more extensive thermal stress propagated to the non-exposed column and the pier cap. As discussed previously, residual stresses and strains resulting from this analysis stage were exported the following impact and blast analysis phase. It is noteworthy that material strength reduction was applied to concrete and steel reinforcement following fire exposure and based on the maximum recorded element temperature, as detailed elsewhere [13,15,16].

#### 3.2.2. Response Under Impact and Blast

**Figure 6:**
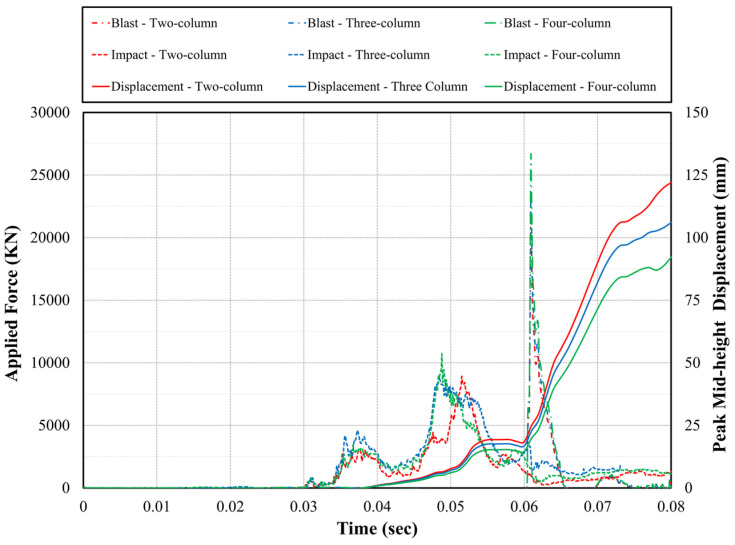
Force and mid-height displacement time histories under impact and air blast.

Force–time history and mid-span column displacement associated with impact and blast loads are depicted in Figure 6. As depicted in this figure, three peak forces were observed during the extreme event combining impact and air blast, including (i) vehicle frame impact at 37 ms; (ii) engine collision 55 ms; and (iii) incident blast wave strike at 62 ms. It is clearly illustrated that force–time histories were not perfectly synchronized across the studied pier configurations. This observation can refer to the variation in lateral displacements caused by fire exposure. This is also due to the discrepancy in structural stiffness piers sustained after being subjected to fire, which in turn influenced their response to the subsequent demands. For instance, the peak engine impact on the two-column pier occurred 2 ms after the peak associated with the four-column pier, indicating that the two-column pier was more susceptible to the initial frame impact and experienced a slightly larger displacement response. Unsurprisingly, and for the same reasons, impact and blast forces increased as the number of pier columns increases. This can be attributed to the increased structural stiffness associated with more columns. Despite undergoing the highest demand, the four-column pier experienced 25% lower mid-height displacement compared to the two-column pier. This can be attributed to the enhanced load sharing, structural redundancy, and flexural stiffness associated with the four-column pier.

**Figure 7:**
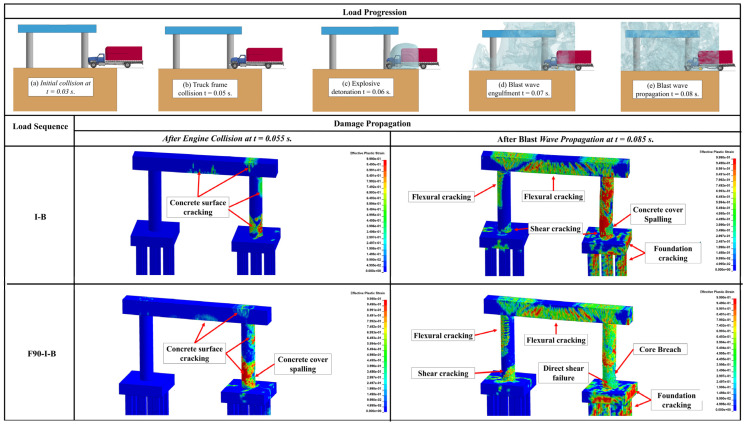
Response of the two-column pier under I-B and F90-I-B.

**Figure 8:**
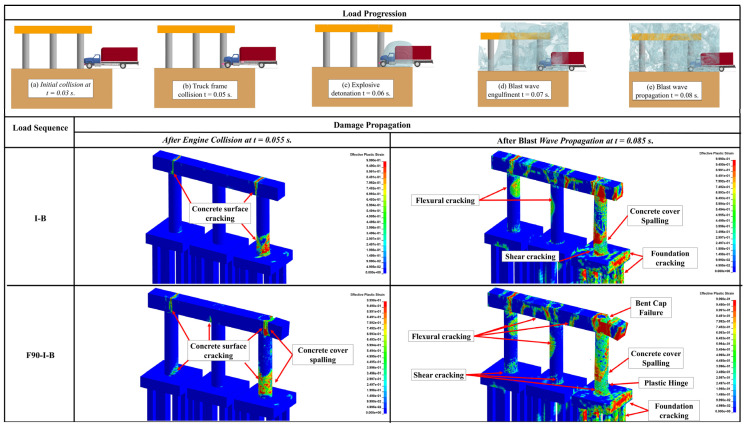
Response of the three-column pier under I-B and F90-I-B.

**Figure 9:**
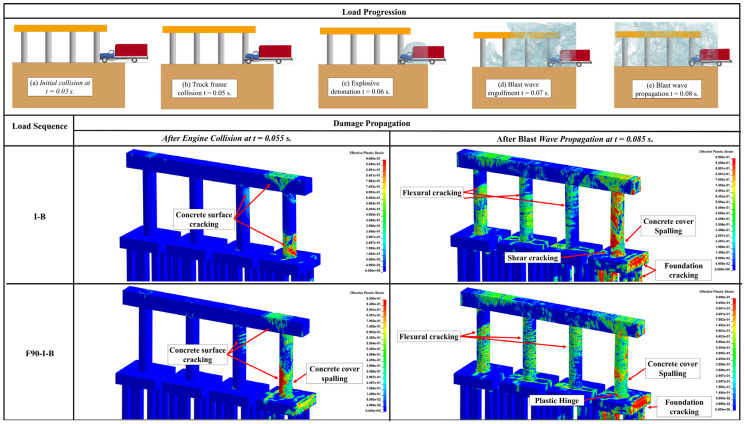
Response of the four-column pier under I-B and F90-I-B.

Unlike the structural response under the quasi-static thermal load, the overall response of RC pier system is governed by the apparent strength increase under the high-loading rates induced by impact and blast loadings leading to reduced ductility. This variation in the mechanical characteristics increases the likelihood of concrete spalling, steel reinforcement rupturing, shear failure, and other brittle failure modes. The structural response of the three studied pier systems when subjected to a post-fire sequential impact and blast is demonstrated in Figure 7, Figure 8 and Figure 9. In these figures, the case that involved impact and blast only (i.e., *I-B*) was taken as the baseline to study the effect of incorporating fire in conjunction with these dynamic events (i.e., *I-B-F90*), where “*I*” refers to impact, “*B*” designates blast, and “*F90*” corresponds to the 90 min fire. Furthermore, qualitative responses after the maximum impact load (i.e., engine impact) and the blast wave propagation are compared to the aforementioned loading scenarios. It is worth noting that effective plastic strain represents the damage parameter and is depicted in all heat maps associated with each damage state. As indicated previously, this specific parameter is correlated to the damage intensity in *MAT159* used to model the concrete, with crack formulation corresponding to a value of 1 and 0 referring to no damage [45]. As discussed previously in Section 2.2.1, concrete spalling is also considered by implementing element erosion technique.

Compared to the *I-B* loading condition, Figure 7, Figure 8 and Figure 9 demonstrated that all piers sustained more significant cracking in the region of impact in the case of *F90-I-B* right after the engine impact. It was also observed that *F90-I-B* encompassed more critical shear and flexural cracking at the bases of the non-impacted columns and the pier cap in the two- and three-column piers. Furthermore, a plastic hinge was formulated in the vicinity of the impact zone, which corresponds to concrete spalling and yielding of reinforcement bars, in the two-column pier.

The final damage state after imposing all demands was also examined. Following earlier observations, *F90-I-B* case was deemed more critical compared to *I-B* scenario. Additionally, the two-column pier system was shown to be more vulnerable and will potentially fail under these extreme demands. Particularly, the structural response associated with this pier structure encompassed critical cracking in all columns, extensive concrete spalling, and complete core breach, which is an indication of a direct shear failure. Despite the lower damage intensity, the three-column pier exhibited similar response including crack propagation to the non-impacted columns and pier cap. However, no rupture was noticed in the steel reinforcement indicating relatively better resilience compared to the two-column pier. On the other hand, sacrificial concrete cracking was propagated along the height of all non-impacted columns in the four-column pier. Moreover, concrete spalling was localized only in the impacted column. It should be mentioned that, irrespective of the number of pier columns, concrete cracks were developed in the foundation system. From a practical perspective, complete replacement is deemed necessary for the two-column pier, while the three- and four-column piers are shown to be repairable and could remain in service. This finding highlights the enhanced structural performance when more columns are used due to their improved load sharing capability and the additional resistance and stiffness they provide.

**Figure 10:**
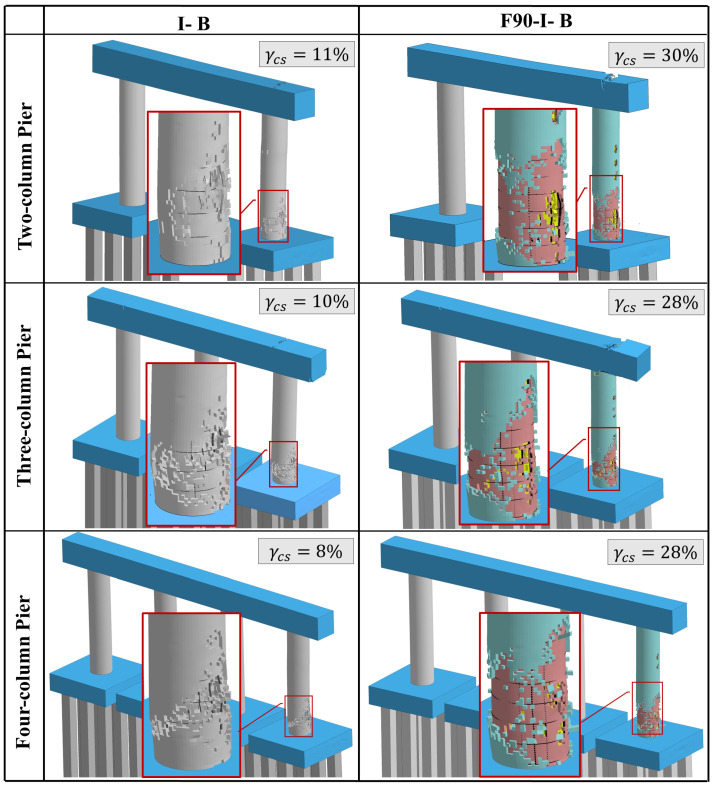
Final damage states under I-B and F90-I-B.

To further visualize the resulting damage, Figure 10 depicts the final damage states of all pier systems under *I-B* and *F90-I-B* loading sequences. In this figure, the volume of spalled concrete ($\gamma_{cs}$) is presented to bring in an additional aspect to the established comparison. Obviously, exposing the impacted column to fire prior to impact and blast resulted in more critical concrete spalling compared to the case that included only impact and blast. This is because fire exposure considerably reduces concrete strength. Additionally, and akin to the previous discussion, the two-column pier was more susceptible to the studied multi-hazard event.

**Figure 11:**
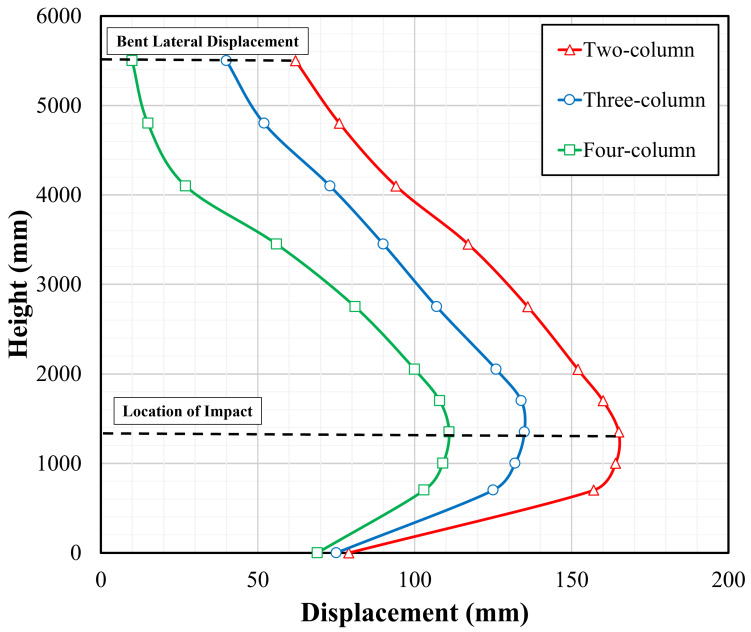
Permanent sets of impacted columns in two-, three-, and four-column pier.

In conjunction with the previous qualitative comparison, lateral displacements associated with impacted pier columns are shown in Figure 11. As demonstrated by this figure, the peak displacement was observed at the impact location. Again, the impacted column within the two-column pier sustained the largest permanent deflection along its height.

**Figure 12:**
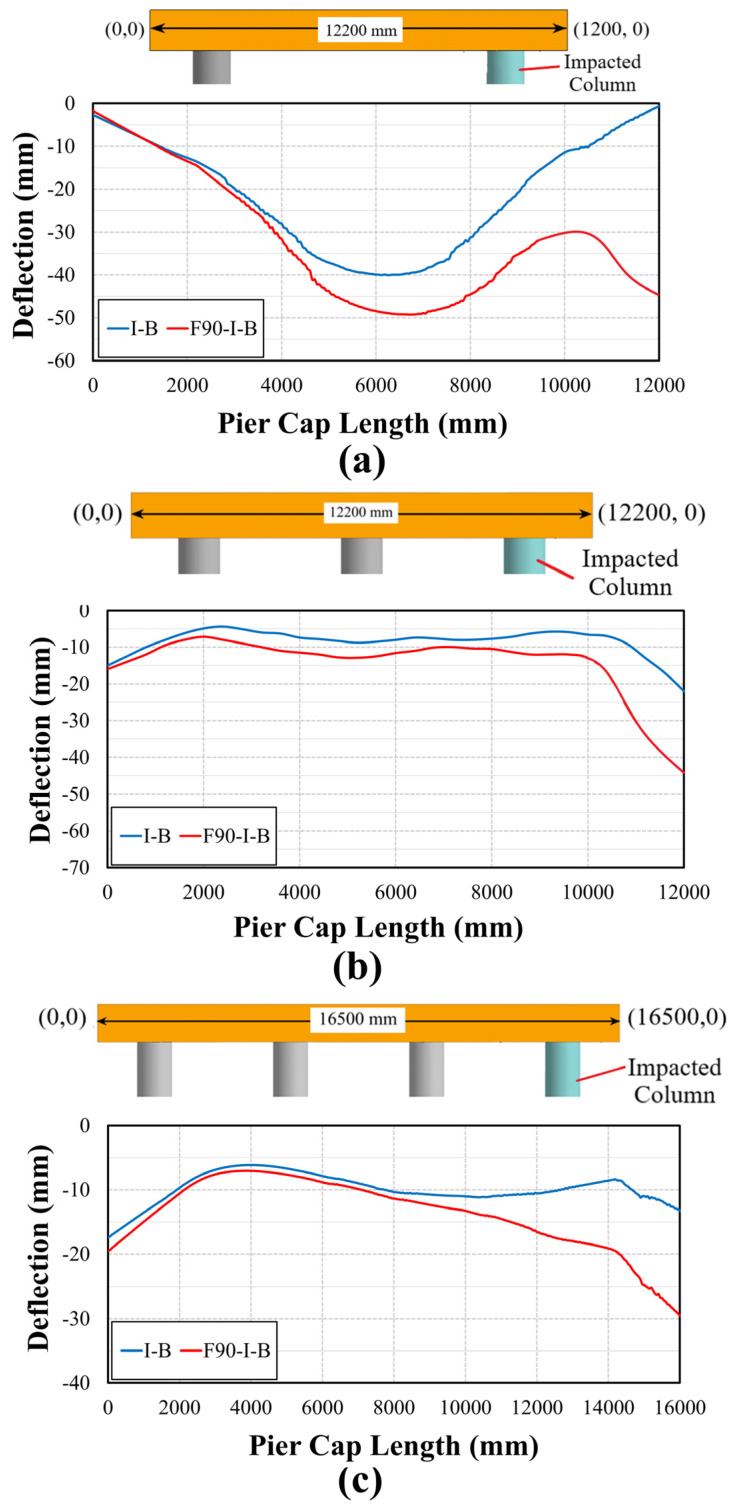
Pier cap deflection: (**a**) two-column pier; (**b**) three-column pier; (**c**) four-column pier.

The deflection of pier caps under *I-B* and *F90-I-B* is illustrated in Figure 12. This figure further emphasizes earlier findings. Compared to *I-B*, a considerably larger deflection occurred above the impacted columns for all piers under *F90-I-B*. Furthermore, it was evident that the failure of the pier cap above the impacted column was expected in the two- and three-column piers. This figure underscores that the number of columns that support the pier cap significantly affected its deflection, with more columns attributed to lower displacement. This observation clearly justifies the recommendation of replacing the two-column pier after such events as the corresponding bridge superstructure sustained extensive deformations.

### 3.3. Response of CFRP-Repaired Two-Column Pier

#### 3.3.1. Retrofit Design

**Figure 13:**
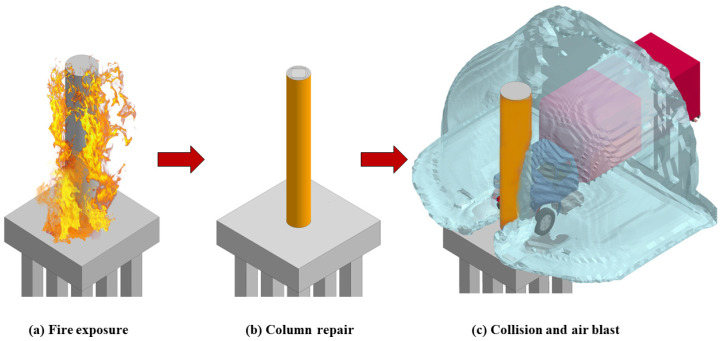
Load sequence and implementation of CFRP retrofit.

The findings presented in the previous sections indicated that the two-column pier configuration largely failed under the imposed extreme demands. Hence, this critical configuration was selected to examine the effectiveness of various CFRP retrofit schemes in improving the structural response and resiliency under post-fire vehicle impact and blast. As shown in Figure 13, the column was repaired after fire exposure and applying the impact and blast loads prior.

**Figure 14:**
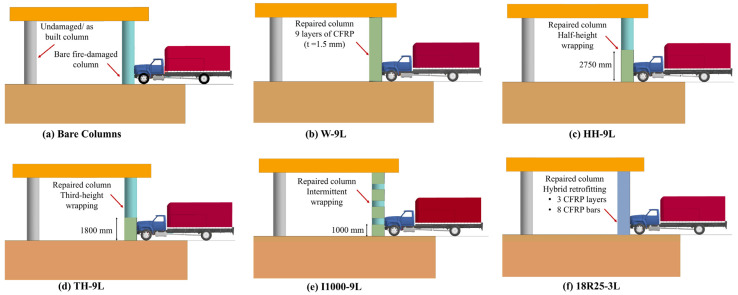
Selected CFRP retrofit scheme.

Following a previous stage in association with this research study that explored the performance of isolated pier columns repaired using multiple CFRP composites [71], two schemes were examined in the current study. Externally bonded (EB) CFRP sheets were used and included (i) wrapping the entire column height; (ii) wrapping the half-column height; (iii) wrapping the bottom third of the column height; and (v) intermittently wrapping along the column height. In this retrofit scheme, an overall CFRP thickness of 1.5 mm was used for all wrapping configurations. On the other hand, a hybrid retrofit scheme utilizes a combination of near surface mounted (NSM) CFRP reinforcing bars and EB CFRP wraps. In this scheme, 18 longitudinal bars of 25 mm diameter and a 0.5 mm thick wrap were used. The studied CFRP retrofit schemes and acronyms associated with each configuration are illustrated in Figure 14.

#### 3.3.2. Structural Response

Generally, utilizing the selected CFRP retrofitting schemes enhances structural performance and the capability to dissipate the energy of impact and blast. Particularly, wrapping the columns provides additional confinement and limits the crack propagation and concrete cover spalling. Additionally, incorporating NSM CFRP bars contributed to improved flexural resistance under the lateral impact and blast demands. Combining both techniques resulted in effectively distributing the imposed loads and enhancing the pier performance.

**Figure 15:**
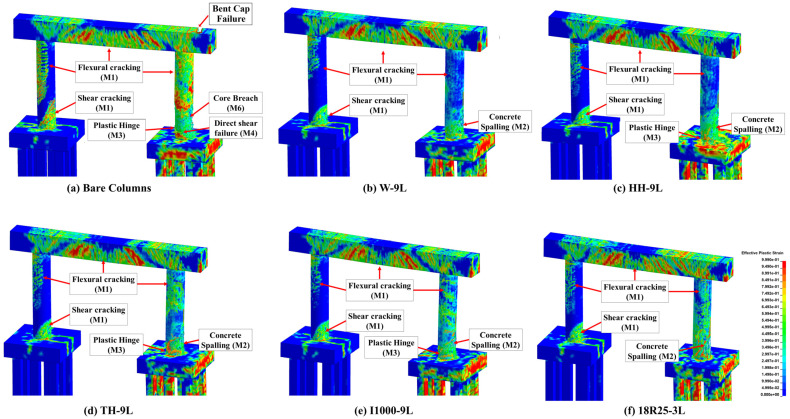
Damage propagation and bare and CFRP-retrofitted piers.

The effectiveness of the selected CFRP retrofit schemes was investigated through qualitative and quantitative comparisons between the performance of bare and repaired piers. Again, an effective plastic strain was used to represent damage propagation in all studied scenarios, as shown in Figure 15. As shown in this figure, the overall damage was considerably mitigated when CFRP repairs were implemented. Particularly, all repair schemes were deemed effective in reducing the intensity of the flexural and shear cracking initiated along the height of the impacted and non-impacted columns, with no column core breach observed. It was also noted that localized plastic hinges formulated in the vicinity of impact location when partial or intermittent wrapping techniques (i.e., *HH-9L*, *TH-9L*, and *I1000-9L*) were employed. On the other hand, the columns repaired using hybrid retrofit scheme (i.e., *18R25-3L*) exhibited more critical concrete surface cracking. These observations indicated that wrapping the full column height (i.e., *W-9L*) showed prominent performance enhancement and damage mitigation.

**Figure 16:**
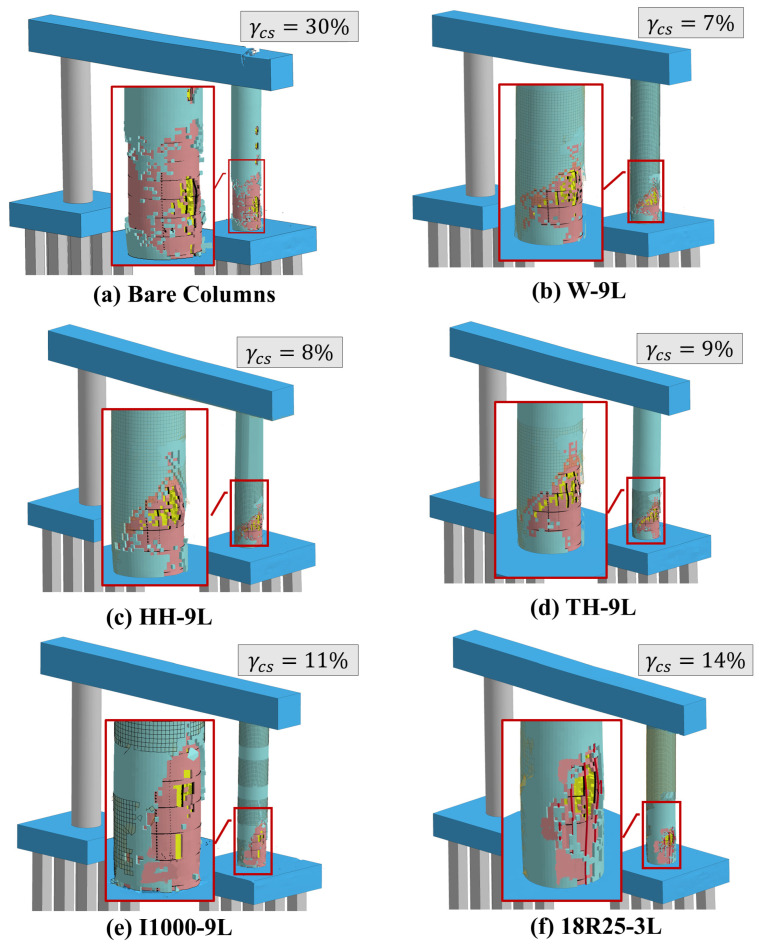
Final damage states under I-B and F90-I-B of bare and CFRP-repaired columns.

Similar to the previous assessment strategy, final damage states and corresponding spalling volumes are depicted in Figure 16. Again, it is obvious that all retrofit schemes significantly mitigated concrete spalling, and a more prominent reduction was observed when the full column height was wrapped. In contrast, the columns retrofitted with the hybrid technique showed an increased level of spalling relative to the other cases investigated. Taking into account that the predominant spalling was concentrated in the impact region and that minimal reinforcement buckling was identified, it is proposed that only minor repairs will be necessary to re-establish the functionality of all piers. It is unequivocally acknowledged that inspectors must assess the levels of deterioration subsequent to impact or blast events through visual inspection, nondestructive testing, and analyses consistent with the *AASHTO Manual for Condition Evaluation and Load Resistance Factor Rating (LRFR) of Highway Bridges* as well as Federal Highway Association (*FHWA*) regulations in order to ascertain the feasibility of any required repairs [85,86].

**Figure 17:**
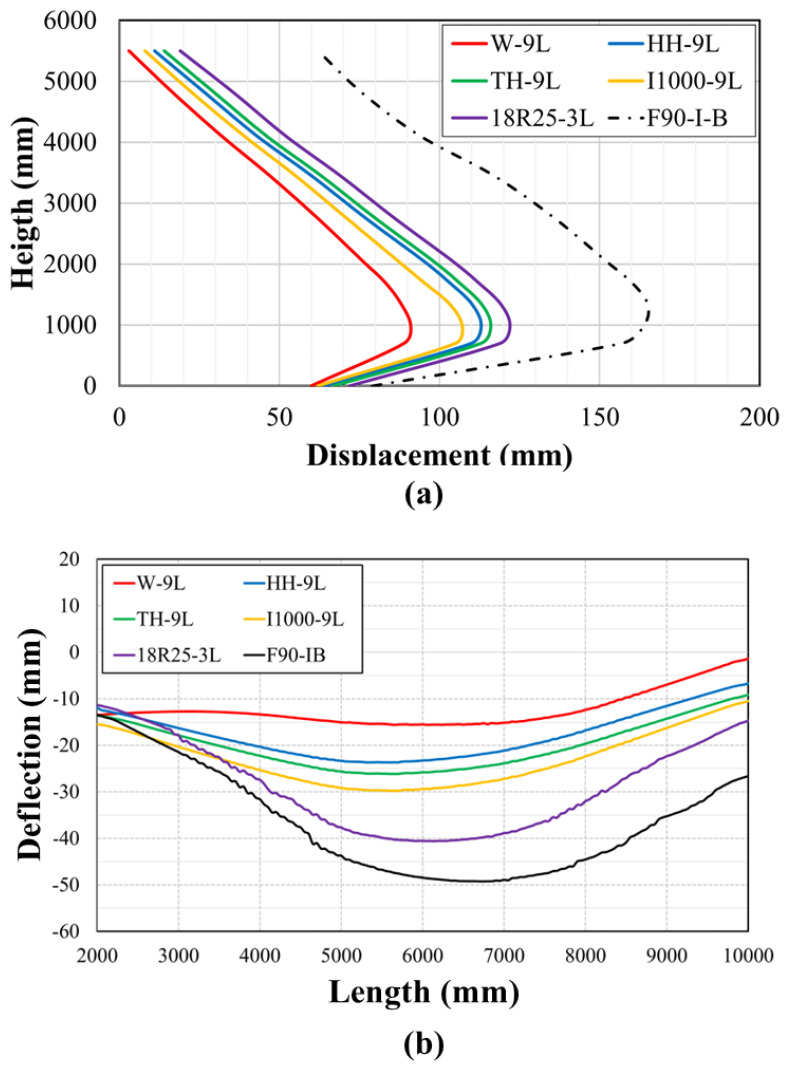
Final displacement: (**a**) impacted column; (**b**) pier cap.

Figure 17 illustrates the final deflection of the impacted columns and pier caps, both for bare and retrofit conditions. Results shown in this figure demonstrate that all employed techniques led to a substantial decrease in lateral displacements. While it became apparent that a marginal difference in peak displacements was noted for columns retrofitted with *W-9L*, all wrapping schemes were deemed effective in reducing column and cap deflections. In addition to the previous findings, this observation highlights the efficacy of wrapping the lower third of pier columns and presents a more cost-effective solution for professionals aiming to minimize damage and deformation. Nevertheless, it is crucial to acknowledge that, while the hybrid retrofitting method resulted in a significant reduction in lateral displacements, its capacity to diminish spalling and cracking was somewhat restricted. Consequently, the adoption of the investigated hybrid retrofitting approach may not be prudent due to the corresponding costs.

## 4. Summary and Conclusions

Following earlier research stages, this study encompasses investigating the performance of multi-column bridge piers under a critical load combination of post-fire medium truck collision and a subsequent air blast previously identified [13,15,16]. The study considered two-, three-, and four-column piers designed in accordance with AASHTO LRFD bridge design requirements [10,84]. The performance of these structural systems was qualitatively and quantitatively assessed based on accumulated damage, load propagation, and permanent sets of deformations. Piers subjected to only impact and blast were taken as reference to examine the influence of fire exposure. The effectiveness of two in-situ retrofit schemes that utilized various CFRP composites and configurations was also studied. The study findings indicate the following:

1. Exposing one column to fire caused substantial damage in the impacted and non-impacted columns irrespective of the number of pier columns.
2. The two-column pier was susceptible to the imposed demands withstanding sever damage and complete replacement deemed to be necessary.
3. While the three-column pier sustained less critical damage than the two-column pier, extensive repairs are still needed to restore its design capacity and integrity. On the other hand, the four-column pier could potentially continue in service while being repaired given the localized damage.
4. Generally, all proposed CFRP retrofit schemes effectively reduced the damage intensity. However, wrapping the full column height was the optimal scheme compared to all other cases.
5. The hybrid retrofit scheme, which included NSM CFRP bars with EB wrapping, was the least effective technique. Given that a 0.5 mm thick CFRP sheet was used in this scheme, this finding highlight that the thickness of CFRP composites has more influence on performance enhancement compared to the reinforcement ratio of CFRP bars.
6. From a practical perspective, wrapping the bottom half or third of column height was shown to be more economically justified given the adequate level of damage mitigation they offer.

## Figures and Tables

**Table 1:** Concrete and steel reinforcement properties.

Material		Parameters		
Concrete	Mass Density	Compressive Strength		Max. Aggregate Size
	$2380\;Kg/m^{3}$	28 MPa		19 mm
Steel	Mass Density	Elastic Modulus	Poisson’s Ratio	Yield Strength
	$2380\;Kg/m^{3}$	200 GPa	0.3	475 MPa

**Table 2:** Soil properties.

Parameter	Specific Gravity	Bulk Modulus	Shear Modulus	Friction Angle	Cohesion Coefficient
**Value**	2.65	146 MPa	56 MPa	35°	$5\times 10^{-6}$ MPa

**Table 3:** Air properties and Linear Polynomial EOS parameters.

Material	Air	Linear Polynomial EOS Parameter		
**Parameter**	Mass Density	E_*o*. *air*_	$C_{0}-C_{3}$ $,\;\mathrm{and}\;C_{6}$	$C_{4}$ $\;\mathrm{and}\;C_{5}$
**Value**	$1.29\times 10^{-3}\;Kg/{mm}^{3}$	0.25 MPa	0	0.4

**Table 4:** TNT explosive properties and JWL EOS parameters.

Material	TNT Explosive			JWL EOS Parameter					
**Parameter**	Mass Density	Detonation Velocity	Chapman–Jouget Pressure	$E_{o,v}$	A	B	$R_{1}$	$R_{2}$	ω
**Value**	$1.63\times 10^{-6}$	6930 m/s	21 GPa	7 GPa	371.2	3.23	4.15	0.95	0.3

**Table 5:** CFRP properties.

Parameter	Mass Density	Poisson’s Ratio	Sheet Thickness	Long. Elastic Modulus	Trans. Elastic Modulus
**Value**	$1.8\;g/{cm}^{3}$	0.021	0.167 mm	200 GPa	9.65 GPa
**Parameter**	Long. Tensile Strength		Trans. Tensile Strength		Ultimate Tensile Strain
**Value**	3850 MPa		57 MPa		1.91%

## Data Availability

Data are available on request due to restrictions. The data presented in this study are available on request from the corresponding author. The data are not publicly available because this study is part of an ongoing, large research project.
